# ACE Inhibitor/ARB Therapy and Other Risk Factors for COVID-19 Infection in Elderly Hypertensive Patients: Sub-Group Analysis Based on a Single-Center, Retrospective, Observational Study in Japan

**DOI:** 10.3390/pharmacy14010022

**Published:** 2026-02-02

**Authors:** Kazuhiro Furumachi, Akari Higuchi, Tatsuki Kagatsume, Mariko Kozaru, Tsutomu Nakamura, Etsuko Kumagai, Keiko Hosohata

**Affiliations:** 1 Department of Nephrology, Kenwakai Hospital, 1936, Kanaenakadaira, Iida 395-0801, Nagano, Japan; k-furumati@kenwakai.or.jp (K.F.); e-kumagai@kenwakai.or.jp (E.K.); 2 Education and Research Center for Clinical Pharmacy, Faculty of Pharmacy, Osaka Medical and Pharmaceutical University, 4-20-1, Nasahara, Takatsuki 569-1094, Osaka, Japantsutomu.nakamura@ompu.ac.jp (T.N.); 3 Department of Drug Evaluation and Informatics, Faculty of Pharmaceutical Sciences, Himeji Dokkyo University, 7-2-1 Kamiohno, Himeji 670-8524, Hyogo, Japan

**Keywords:** hypertensive patients, COVID-19, risk factor, angiotensin-converting enzyme inhibitor, angiotensin II receptor blocker, Japanese

## Abstract

Background: Angiotensin-converting enzyme inhibitors (ACEIs) and angiotensin II receptor blockers (ARBs) are often used in hypertensive patients. Severe acute respiratory syndrome coronavirus 2 (SARS-CoV-2), the virus responsible for the coronavirus disease 2019 (COVID-19) pandemic, binds the ACE2 receptor on the cell surface. This study aimed to identify the risk factors influencing COVID-19 infection in hypertensive patients. Methods: This is a part of a single-center, retrospective, observational study investigating patients ≥ 20 years old at Kenwakai Hospital (Nagano, Japan). COVID-19 was diagnosed by polymerase chain reaction. All patients received antihypertensive drugs. Results: Among 316 patients (mean age, 75.0 ± 13.4 years; men, 55.1%), COVID-19 was diagnosed in 39 (12.3%). Multiple logistic regression analysis after adjustment for age, sex, and smoking status identified increased serum creatinine (Scr) as a significant risk factor for COVID-19 (odds ratio [OR] 1.10; 95% confidence interval [CI] 1.00–1.20; *p* = 0.046). Conversely, lower serum chloride was associated with COVID-19 (OR 0.92; 95% CI 0.85–0.99; *p* = 0.047). There was no significant association between COVID-19 and the use of ACEIs and ARBs. Conclusions: Scr was independently associated with COVID-19 risk, whereas ACEI/ARB use was not associated with COVID-19 risk in Japanese hypertensive patients, suggesting that these users need not discontinue or change their treatment. The study population included a very high proportion of patients with advanced chronic kidney disease, which makes the cohort substantially different from the general hypertensive population. However, our results can help guide targeted treatment strategies, improving patient outcomes in healthcare settings.

## 1. Introduction

Coronavirus disease 2019 (COVID-19), which can be transmitted not only by individuals who have developed symptoms, but also by those who are pre-symptomatic or asymptomatic carriers of the pathogen, is caused by severe acute respiratory syndrome coronavirus 2 (SARS-CoV-2). Typically, COVID-19 shows mild to moderate symptoms, but is severe or life-threatening for approximately 5–10% of patients [1], potentially requiring artificial ventilation and admission to the intensive care unit (ICU). Among COVID-19 survivors who had received ICU treatment, approximately 74% exhibited physical symptoms, approximately 26% exhibited mental symptoms, and approximately 16% exhibited cognitive symptoms [2]. The most prevalent comorbidities of ICU patients with COVID-19 have been identified as diabetes mellitus (DM), hypertension, cerebrovascular disease, and coronary heart disease [3,4]. Hypertension, whether associated with other comorbidities or not, was especially observed to worsen the course of COVID-19 [5].

Concerns have been raised about the potential impact of treatment for hypertension using renin–angiotensin system (RAS) inhibitors [angiotensin-converting enzyme (ACE) inhibitors (ACEIs) and/or angiotensin II type I receptor blockers (ARBs)] on COVID-19 and its outcomes. The expression of ACE2, to which SARS-CoV-2 binds on the surface of the target cell, can be upregulated in tissues by exposure to ACEIs and/or ARBs [6,7]. Despite potential mechanistic differences in ACEIs and ARB, it is possible that such upregulation of ACE2 expression could result in patients becoming more susceptible to COVID-19 infection. However, studies have produced conflicting results, showing increased risk [8,9], decreased risk [10,11], and no association [12,13] with COVID-19 positivity or COVID-19 after ACEI and/or ARB treatment. Moreover, data are still limited, particularly among Japanese patients.

The aim of this study is to explore COVID-19 risk factors, including the use of ACEIs/ARBs, among Japanese hypertensive patients.

## 2. Materials and Methods

### 2.1. Study Design and Data Collection

This retrospective and observational study included patients aged ≥20 years at Kenwakai Hospital (Iida, Japan). The study’s details have been described previously [14,15]. This study was approved by the ethics committees of Kenwakai Hospital (approval no. 2023004), Himeji Dokkyo University (approval no. 24-03), and Osaka Medical and Pharmaceutical University (approval no. 2023-061). The ethics committees waived the need to obtain written informed consent from patients because of the retrospective and anonymous nature of the study. This study was conducted in accordance with the Declaration of Helsinki.

**Figure 1:**
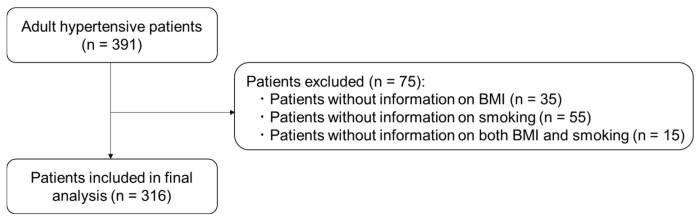
Flow diagram of patient selection.

Electronic medical charts were used to collect data on eligible adult hypertensive patients who visited the hospital between 1 January 2019 and 28 February 2023. Systolic blood pressure (BP) ≥ 140 mmHg, diastolic BP ≥ 90 mmHg, or the use of antihypertensive drugs were defined as hypertension. The Charlson Comorbidity Index was calculated for each patient at time of diagnosis [16]. Complications were scored by Clavien–Dindo classifications. We excluded data from patients lacking information on body mass index (BMI) or smoking status. The remaining patients were then included in the present analyses (Figure 1).

### 2.2. Outcome Definition

In daily practice, COVID-19 was diagnosed by polymerase chain reaction (PCR). The primary outcome was thus defined as COVID-19 infection during the observation period.

### 2.3. Statistical Analysis

Data are presented as mean ± standard deviation (SD), median (intertertile range), or frequency with percentage according to the types and distribution of variables. Differences between groups of normally distributed variables were analyzed by *t* test. Differences between groups of nonparametric variables were analyzed using the Mann–Whitney *U* test. Categorical variables were compared using Pearson’s chi-squared test or Fisher’s exact test. Logistic regression models were used to examine associations between the risk of COVID-19 infection and patient characteristics. Significance was defined as values of *p* < 0.05 (two-tailed). All statistical analyses were performed using SPSS for Windows (ver. 19.0; SPSS, Tokyo, Japan).

## 3. Results

### 3.1. Patient Characteristics

Table 1 shows the characteristics of the study patients. Of the 316 patients, 174 (55.1%) were men. The mean age, BMI, systolic BP, and diastolic BP were 75.0 ± 13.4 years, 22.0 ± 3.9 kg/m^2^, 143.6 ± 23.3 mm Hg, and 78.8 ± 14.5 mmHg, respectively. Of the study patients, 21 (6.6%) were classified as smokers, and 172 (54.4%) were on maintenance dialysis due to chronic kidney disease (CKD). Among the 316 patients, 39 (12.3%) were diagnosed with COVID-19 by PCR-confirmed tests for the SARS-CoV-2 virus.

### 3.2. Risk Factors Associated with COVID-19

As shown in Table 1, no significant differences between groups were identified in age, sex, BMI, smoking status, BP, dialysis status, or Charlson Comorbidity Index between patients infected with COVID-19 and uninfected control patients. Regarding laboratory data, patients infected with COVID-19 exhibited significantly lower levels of lactate dehydrogenase (LDH) (*p* = 0.045), serum chloride (*p* = 0.034), and high-density lipoprotein (HDL) cholesterol (*p* = 0.028), and higher concentrations of Scr (*p* = 0.031), β2-microglobulin (*p* = 0.032), and white blood cells (*p* = 0.048) than uninfected control patients.

Drug therapy was prescribed as follows: antidiabetic drugs, 81 (25.6%); antilipidemic drugs, 132 (41.8%); anticoagulant and antiplatelet drugs, 49 (15.5%); aspirin, 57 (18.0%); dexamethasone, 5 (1.6%); antihypertensive drugs, 316 (100%). At the time of COVID-19 diagnosis, a median of 385 days (25th-75th percentile, 302–486 days) passed from the medication use. There was no significant difference in the use of medications (Table 2).

Notably, there was no significant difference in the use of RAS inhibitors (ACEIs and/or ARBs) between patients infected with COVID-19 and uninfected control patients (51.3% vs. 51.3%). Other antihypertensive drugs were also used in both groups (48.7% vs. 48.7%).

We performed multivariate logistic regression analysis using these statistically significant variables (Table 3). The risk of COVID-19 infection was significantly increased with higher Scr (odds ratio [OR] 1.08, 95% confidence interval [CI] 1.01–1.16; *p* = 0.033). This significance remained after adjustments for possible confounding factors (age, sex, smoking). Conversely, the risk for COVID-19 infection was significantly increased with lower serum chloride (OR 0.92, 95% CI 0.85–0.99; *p* = 0.034) and HDL cholesterol (OR 0.98, 95% CI 0.95–0.998; *p* = 0.036). However, after multivariate adjustment, HDL cholesterol was not significantly associated with the risk of COVID-19 infection.

## 4. Discussion

This is the first study to demonstrate that high Scr was a risk factor for COVID-19 infection after adjusting for possible confounding factors (age, BMI, and smoking) in Japanese hypertensive patients. Moreover, the risk of COVID-19 infection increased significantly along with decreased serum chloride, and this significant association remained in a multivariable logistic regression model. Importantly, the use of ACEIs and/or ARBs was not significantly associated with the risk of COVID-19 infection. Thus, our results provide evidence of a low impact of RAS inhibitor use on the susceptibility to COVID-19 infection among Japanese hypertensive patients.

Our results revealed that high Scr was significantly associated with COVID-19 infection. Several studies have reported disorders in the immune system of CKD patients [17,18]. The progressive loss of renal function is associated with lymphopenia in specific T-cell subsets [16]. Diminished T-cell activation could lead to a higher susceptibility to infectious complications in CKD patients. In addition, significantly decreased numbers of B lymphocytes were observed in peripheral blood from patients with end-stage renal disease [19]. Stage 5 CKD, as the final stage of CKD, was classified as occurring when patients had an eGFR less than 15 mL/min/1.73 m^2^ regardless of the need for renal replacement therapy. We found that patients infected with COVID-19 showed higher SCr than uninfected control patients. In our results, 66.7% of COVID-19 patients showed eGFR <15 mL/min/1.73 m^2^. One possibility is that these patients have immune system disorders. In addition, patients in our cohort receiving dialysis accounted for 64.1% of COVID-19 patients. A previous study showed that the number of CD4+ and CD8+ cells is diminished in hemodialysis patients, suggesting that hemodialysis may trigger T-lymphocyte apoptosis [20]. In our results, hypertensive patients with low serum chloride were more susceptible to COVID-19 infection. Serum chloride, which is found predominantly in extracellular fluid, is a crucial electrolyte [21]. Valga et al. [22] report an association of hypochloremia with COVID-19 infection. We suggest that serum chloride levels are monitored to assess the progression of infectious diseases, including COVID-19, in hypertensive patients.

The present study provides another potentially important finding that the use of RAS inhibitors did not increase susceptibility to COVID-19 infection among Japanese hypertensive patients. First of all, spike proteins coat the surface of SARS-CoV-2, and a subdomain of the spike protein directly binds to ACE2 to allow delivery of the viral genome [23]. Of note, ACEIs and ARBs have been shown to increase ACE2 enzymatic activity and expression of its protein and gene [24,25]. ACE2 is abundantly expressed in the lungs, heart, and other tissues [26]. The use of ACEIs and/or ARBs has thus been hypothesized to enhance susceptibility to COVID-19 infection. In Japan, it is estimated that approximately 43 million people suffer from hypertension, showing approximately one in three Japanese individuals exhibit hypertension. Importantly, ACEIs and/or ARBs are widely used in Japanese hypertensive patients [27]. Indeed, our study cohort showed that almost half of patients used ACEIs and/or ARBs (51.3%). However, our study found no significant relationship between the use of ACEIs and/or ARBs and COVID-19 infection in Japanese hypertensive patients. This is consistent with a population-based study that showed the use of ACEIs and ARBs was more frequent among COVID-19 patients than among controls, but found no evidence for ACEIs or ARBs affecting the risk of COVID-19 [28]. Our results suggest no need to discontinue or change treatment. Importantly, COVID-19 is a multisystemic disease that affects several organs. Hypertension is often associated with other pathologies (metabolic disorders) which have a negative impact on the outcome of COVID-19 patients [29]. The correct treatment of arterial hypertension with ACEI is very important, especially in hypertensive patients with high to very high cardiovascular risk [30].

Several limitations to this study must be kept in mind when interpreting the results. First, this study used a relatively small sample. Second, this was a real-world observational study, so the control and case groups were not equivalent. Third, the research institution involved is a dialysis center, and more than 50% of both the COVID-19 group and the non-infected group comprised dialysis patients. The normal reference value for eGFR is eGFR ≥ 90 mL/min/1.73 m^2^, but only 4.1% of the total study population fell within this range. Thus, the patient population in this study had low renal function. Fourth, in our results, range of data are presented as an intertertile because the number of data in COVID-19 group was small. Each individual value carries significant weight, and adding just one more data point could cause the intertertile range to change dramatically. Finally, all data were from one hospital in Japan, so the findings may not be generalizable to other populations.

## 5. Conclusions

This study demonstrated that the risk of COVID-19 infection significantly and independently increased with high Scr and low serum chloride, whereas no significant association was identified between ACEI/ARB use and COVID-19 infection in Japanese hypertensive patients. In our results, ACEI/ARB use was not a significant contributor to the infection in hypertensive patients, suggesting no need to discontinue or change treatment. The study population included the very high proportion of patients with advanced CKD, which makes the cohort substantially different from the general hypertensive population. Further studies in larger populations are needed to confirm our findings and to assess the utility of risk factors for COVID-19 infection in Japanese hypertensive patients.

## Figures and Tables

**Table 1:** Demographic and clinical characteristics.

Variables	Total (*N* = 316)	COVID-19 (+)	COVID-19 (−)	*p*
		*N* = 39 (12.3%)	*N* = 277 (87.7%)	
Age, years	75.0 ± 13.4	75.0 ± 13.9	75.0 ± 13.3	0.94
Men, *n* (%)	174 (55.1)	26 (66.7)	148 (53.4)	0.12
BMI, kg/m^2^	22.0 ± 3.9	21.7 ± 3.8	22.1 ± 3.9	0.56
Smoking, *n* (%)	21 (6.6)	1 (2.6)	20 (7.2)	0.49
Systolic BP, mmHg	143.6 ± 23.3	142.2 ± 23.2	143.8± 23.4	0.99
Diastolic BP, mmHg	78.8 ± 14.5	78.8 ± 15.6	78.8 ± 14.4	0.86
Dialysis, *n* (%)	172 (54.4)	25 (64.1)	147 (53.1)	0.20
Charlson Comorbidity Index	6 (6–7)	6 (6–7)	6 (6–7)	0.80
Laboratory data				
Serum Zn, μg/dL	65.0 (60.0–71.0)	66.0 (63.0–68.0)	65.0 (60.0–71.0)	0.52
Total protein, g/dL	6.4 (6.1–6.6)	6.4 (6.0–6.5)	6.4 (6.1–6.7)	0.36
T-bil, mg/dL	0.50 (0.40–0.60)	0.45 (0.40–0.57)	0.50 (0.40–0.60)	0.20
AST, IU/L	17.0 (14.0–19.7)	13.0 (11.0–17.0)	17.0 (14.0–20.0)	0.07
ALT, IU/L	12.0 (10.0–15.0)	12.0 (10.0–14.0)	12.0 (10.0–16.0)	0.52
LDH, IU/L	194.0 (177.0–219.7)	177.5 (164.0–194.7)	197.0 (180.0–220.7)	0.045 *
Uric acid, mg/dL	6.7 (5.9–7.5)	7.0 (6.4–7.7)	6.7 (5.7–7.5)	0.26
BUN, mg/dL	41.3 (22.6–55.9)	58.3 (27.2–64.1)	40.1 (22.3–53.5)	0.06
Scr, mg/dL	6.6 (1.0–9.1)	8.2 (2.9–11.0)	5.9 (1.0–9.0)	0.031 *
eGFR, mL/min/1.73 m^2^				0.59
≥90	13 (4.1)	2 (5.1)	11 (4.0)	
60 to 89	59 (18.7)	5 (12.8)	54 (19.5)	
30 to 59	59 (18.7)	5 (12.8)	54 (19.5)	
15 to 29	10 (3.2)	1 (2.6)	9 (3.2)	
<15	175 (55.4)	26 (66.7)	149 (53.8)	
Serum sodium, mEq/L	140.0 (139.0–141.0)	139.0 (138.0–141.0)	140.0 (139.0–141.0)	0.098
Serum potassium, mEq/L	4.4 (4.1–4.6)	4.5 (4.2–4.6)	4.3 (4.1–4.6)	0.17
Serum chloride, mEq/L	104.0 (102.0–106.0)	103.0 (101.0–104.3)	105.0 (102.0–106.0)	0.034 *
Total cholesterol, mg/dL	158.5 (146.0–174.0)	158.5 (145.0–169.0)	158.5 (146.3–174.7)	0.46
Triglyceride, mg/dL	101.0 (83.0–134.0)	108.0 (87.3–129.0)	98.0 (82.0–134.0)	0.59
HDL cholesterol, mg/dL	50.0 (43.0–59.0)	45.0 (37.7–53.7)	51.0 (44.0–60.0)	0.028 *
LDL cholesterol, mg/dL	92.0 (81.0–103.0)	95.0 (83.7–109.0)	90.5 (81.0–103.0)	0.48
β_2_-microglobulin, mg/L	26.2 (24.5–28.9)	27.9 (26.4–30.8)	25.8 (24.1–28.1)	0.032 *
Plasma glucose, mg/dL	127.0 (111.0–140.0)	128.0 (121.3–148.3)	126.0 (111.0–139.0)	0.27
BNP, pg/mL	81.4 (46.5–165.8)	105.7 (63.4–225.2)	80.3 (46.0–160.4)	0.23
WBC, ×10^3^/μL	5.7 (5.1–6.5)	6.8 (5.8–7.1)	5.6 (5.0–6.3)	0.048 *
RBC, ×10^6^/μL	3.7 (3.4–4.0)	3.7 (3.4–3.9)	3.7 (3.4–4.0)	0.70
Hemoglobin, g/dL	11.4 (10.8–12.1)	11.3 (10.7–11.8)	11.4 (10.8–12.2)	0.41
Hct, %	34.2 (33.0–36.2)	36.7 (34.7–37.1)	34.1 (32.9–36.0)	0.14
PLT, ×10^3^/μL	200.5 (173.0–232.0)	228.0 (204.7–242.0)	197.0 (172.0–230.0)	0.18

Data are presented as mean ± SD, median (intertertile range), or number (percentage). Statistical significance was set at *p* < 0.05. *, *p* < 0.05. Abbreviations: ALT, alanine transaminase; AST, aspartate transaminase; BMI, body mass index; BNP, brain natriuretic peptide; BP, blood pressure; BUN, blood urea nitrogen; eGFR, estimated glomerular filtration rate; Hct, hematocrit; HDL, high-density lipoprotein; LDH, lactate dehydrogenase; LDL, low-density lipoprotein; PLT, platelets; RBC, red blood cells; Scr, serum creatinine; SD, standard deviation; T-Bil, total bilirubin; WBC, white blood cells.

**Table 2:** Baseline drug treatment.

Variables	Total (*N* = 316)	COVID-19 (+)	COVID-19 (−)	*p*
		*N* = 39 (12.3%)	*N* = 277 (87.7%)	
Drugs other than antihypertensive drugs				
Antidiabetic drugs, *n* (%)	81 (25.6)	11 (28.2)	70 (25.3)	0.69
Antilipidemic drugs, *n* (%)	132 (41.8)	17 (43.6)	115 (41.5)	0.81
Anticoagulant and antiplatelet drugs, *n* (%)	49 (15.5)	7 (17.9)	42 (15.2)	0.65
Aspirin, *n* (%)	57 (18.0)	10 (25.6)	47 (17.0)	0.19
Dexamethasone, *n* (%)	5 (1.6)	1 (2.6)	4 (1.4)	0.49
Antihypertensive drugs				
RAS inhibitor, *n* (%)	162 (51.3)	20 (51.3)	142 (51.3)	0.998
Others, *n* (%)	154 (48.7)	19 (48.7)	135 (48.7)	0.998

Data are presented as number (percentage). Statistical significance was set at *p* < 0.05. Abbreviations: RAS, renin–angiotensin system.

**Table 3:** Adjusted odds ratios for COVID-19 infection.

Variables	Unadjusted		Adjusted	
	Odds Ratio (95% CI)	*p*	Odds Ratio (95% CI)	*p*
LDH, IU/L	0.99 (0.98–1.00)	0.059	0.99 (0.98–1.00)	0.58
Scr, mg/dL	1.08 (1.01–1.16)	0.033 *	1.10 (1.00–1.20)	0.046 *
Serum chloride, mEq/L	0.92 (0.85–0.99)	0.034 *	0.92 (0.85–0.99)	0.047 *
HDL cholesterol, mg/dL	0.98 (0.95–0.99)	0.036 *	0.98 (0.95–1.00)	0.057
β2-microglobulin, mg/dL	1.06 (0.99–1.12)	0.095	1.06 (0.99–1.13)	0.13
WBC, ×10^3^/μL	1.01 (0.99–1.02)	0.47	1.01 (0.99–1.02)	0.399

Data are adjusted for age, sex, and smoking status. Statistical significance was set at *p* < 0.05. *, *p* < 0.05. Abbreviations: CI, confidence interval; HDL, high-density lipoprotein; LDH, lactate dehydrogenase; Scr, serum creatinine; WBC, white blood cells.

## Data Availability

All data relevant to the study are included in the article.

## Abbreviations

The following abbreviations are used in this manuscript:

ACEI	angiotensin-converting enzyme inhibitor
ALT	alanine transaminase
ARB	angiotensin II receptor blocker
AST	aspartate transaminase
BMI	body mass index
BNP	brain natriuretic peptide
BP	blood pressure
BUN	blood urea nitrogen
CI	confidence interval
COVID-19	Coronavirus disease 2019
eGFR	estimated glomerular filtration rate
Hct	hematocrit
HDL	high-density lipoprotein
LDH	lactate dehydrogenase
LDL	low-density lipoprotein
PCR	polymerase chain reaction
PLT	platelets
RAS	renin–angiotensin system
RBC	red blood cells
Scr	serum creatinine
T-Bil	total bilirubin
WBC	white blood cells
